# Person, Place, and Time Effects on Cognitive Function Among Older People in Taiwan

**DOI:** 10.1093/geroni/igab046.2632

**Published:** 2021-12-17

**Authors:** Hui-chuan Hsu, Chyi-Huey Bai

**Affiliations:** Taipei Medical University, Taipei, Taipei, Taiwan (Republic of China)

## Abstract

Purpose: Individual’s factors across time or combined with area characteristics related to cognitive function for older people have been widely explored, but little research examined person, place, and time effects altogether. The purpose of this study was to examine the effects of individuals, cities, and time on older people’s cognitive function in Taiwan. Methods: A nation-representative longitudinal individual data were from Taiwan Longitudinal Survey on Aging (TLSA) 1999-2015 panel data (analysis sample n=6349 persons, observations=12042). Cognitive function was scored 0-19. Individual’s factors included demographics, health conditions and health behaviors, mental health and stress, social support and social participation, etc. Eleven city-level indicators were based on 22 cities and data were from the government open data sources. Mixed linear modeling analysis was applied. Results: Better cognitive function was significantly related to individuals’ working, ethnicity, younger age, better education level, better self-rated health, less psychological stress, receiving more emotional support, having higher economic satisfaction at the intercept. Sex, ethnicity, age, education, self-rated health, physical function, and social connectedness were significant at the time slope. When controlling for individuals’ factors, population density and green land were significant at the intercept and at the time slope. Interactions of individual- and city-level factors were not significant. Discussion: Individual’s social participation and social support are protective factors of cognitive function for older adults. And an age-friendly environment providing appropriate cognitive stimulation and chances of social participation may be beneficial for cognitive function.

